# Fatigue during Long-Haul Flights of Different Crew Compositions under Exemption from Layover and Flight Time during COVID-19

**DOI:** 10.3390/ijerph192013567

**Published:** 2022-10-19

**Authors:** Jingqiang Li, Yanru Zhou, Xining Zhang, Tianchen Fan

**Affiliations:** 1 Safety Science and Engineering College, Civil Aviation University of China, No. 2898 Jinbei Road, Dongli District, Tianjin 300300, China; 2 Research Institute of Civil Aviation Safety Science, Civil Aviation University of China, No. 2898 Jinbei Road, Dongli District, Tianjin 300300, China

**Keywords:** intercontinental flights, crew composition, sleep, alertness, fatigue, COVID-19

## Abstract

Pilot fatigue and alertness are critical for civil aviation safety. Intercontinental pilots are more prone to fatigue and sleepiness due to jet lag, prolonged workdays, and disrupted rhythms. The Civil Aviation Administration of China excused enlarged flight crews from mandatory layovers and reimposed flight duration restrictions during COVID-19. This study investigates the sleep quality and attentional performance of pilots on intercontinental flights. The fifteen pilots who performed intercontinental flights in different crew compositions wore a body movement recorder, which has been proven to accurately estimate sleep duration and sleep efficiency. The crew’s attentional performance and self-report were monitored at specified flight phases. In conclusion, the larger crews slept longer and more efficiently on board, particularly pilots in charge of takeoff and landing responsibilities. Crews on four-pilot layover flights were more alert before the takeoff of the inbound flights than exempt flights, but there was no significant difference towards the end of the mission. The new long-haul flight organization did not result in fatigue or decreased attention in the pilots. This study expands on the research by validating a novel intercontinental flight operation model under the COVID-19 scenario and highlighting critical spots for future fatigue management in various crew compositions.

## 1. Introduction

The alertness of the pilot ensures flight safety and that duties are performed correctly. International flight crews usually need work and activities at the window of circadian low (WOCL) when people feel fatigued [1]. Sleep loss and drowsiness contribute to accidents and impaired work performance significantly [2], especially affecting pilot alertness on ultra-long and long-haul flights [3]. Individual attention consists of four components: selective attention, phasic alertness, tonic alertness, and sustained attention, all of which need to be maintained at a suitable level to ensure a safe flight [4]. Selective attention (SA) refers to the differential processing of concurrent sources of information [5]. The “blindness” phenomenon occurs in groups with poor selective attention, which could pose a safety risk to flying. The psychological concept is inattentional blindness, where people concentrate on a task while ignoring an unexpected stimulus [6]. Sustained attention (SUA) helps people sustain performance on tasks over extended periods [7]. Flying an aircraft requires the crew to concentrate for a prolonged duration, so sustained pilot attention during long missions is a prerequisite for safe flying.

Adequate and efficient sleep is essential for the alertness, health, and biorhythm stability of pilots. Although the regulations set limits on flight and rest time, the study found that pilots continued to report high levels of fatigue with significant sleep problems [8]. Fatigue and sleep problems are also strongly related to stress and mental health. The COVID-19 pandemic has rapidly spread across the globe, contributing to anxiety, financial stress, fear, etc. The direction of flight and the number of time zones crossed can have an impact on pilots’ sleep–wake cycle, and there is a significant difference between layover sleep and before-flight sleep [9]. Furthermore, jet lag syndrome caused by rapid travel across time zones can desynchronize the body’s internal rhythm, making it difficult to quickly adapt to the time at the destination; this often manifests as insomnia at night and daytime sleepiness. Pilots able to sleep two nights during layovers appear to provide more adequate opportunities for recovery than one night [10].

Before the COVID-19 outbreak, international flights with four-pilot crews, after arriving at a destination, must disembark and rest at a hotel for a minimum of 10 consecutive hours before completing the return leg of the trip. After the COVID-19 outbreak, the Chinese exemption policy allowed some flight crews exempt from mandatory layover and flight times by enlarging the crew size to reduce the risk of COVID-19 infection. For such exempt flights, the expanded crew (six or eight pilots) could complete a direct intercontinental round-trip flight. On exempt flights, the front row of the business class is used as a separate rest area for pilots, in addition to the rest area within the aircraft. Extended flight time (workday duration longer than 16 h) may lead to extended periods of wakefulness, greater acute sleep loss, or accumulating task fatigue time [11]. However, the avoidance of cross-time zone layovers prevents the crew from jet lag disrupting their biorhythms [12]. Theoretically, a larger crew size could receive a longer in-flight rest period, thus increasing their alertness. Therefore, this study proposes the following hypotheses:

**Hypothesis** **1** **(H1).** *The new intercontinental flight operation model in which the number of pilots was increased to extend working time could relieve pilot fatigue and ensure alertness*.

**Hypothesis** **2** **(H2).** *Poorer sleep quality during layovers abroad than before flight and circadian rhythm disturbances are more likely to occur on non-exempt flights than exempt flights*.

Additionally, the rotation sequence for different crew compositions has also changed under the exemption policy. For a four-pilot crew, one set (two pilots) is responsible for takeoff and landing and part of the cruising stage, and the other set performs the main cruise phase. For a six-person crew, one set of the crew is usually responsible for takeoff and landing, and the other two sets perform the cruise phase of the flight of round trips. For eight-pilot crews, the first crew set is responsible for the takeoff and landing period of outbound, and the second set is responsible for takeoff and landing of inbound, while the remaining pilots are on duty during cruise. In flight missions, pilots need to integrate and monitor multiple instrumentation information to make accurate judgments and predictions about the flight environment. A study has highlighted the increase in heart rates that occurs in pilots during flight and especially during takeoffs and landings, which were attributed to workload and to psychological or emotional stress [13]. Excessive workload might cause rapid fatigue and increased errors in individuals, resulting in reduced operational levels and even human-caused accidents [14]. During long-term cruise duties, the tedious task of supervision requires the crew to maintain sustained attention, failing which they may fall into mind-wandering or microsleep [15,16]. Several studies have found that the human body is at WOCL between 3 and 6 a.m. when sleepiness is the most obvious [17,18]. Thus, for intercontinental pilots, the possibility of errors and accidents is significantly increased at those times; however, because intercontinental flights are long, pilots cannot avoid working during the WOCL, and we posit the following predictions:

**Hypothesis** **3** **(H3).** *Pilots are more fatigued, and their attentional performance is reduced at the end of the shift compared to pre-duty, especially for takeoff and landing*.

**Hypothesis** **4** **(H4).** *Pilots in the takeoff and landing phases of flights during the WOCL have worse attentional performance and feel more fatigue*.

There has been little study of this measure, and it is worthwhile to conduct and in-depth examination on how many additional pilots can operate exempt flights to ensure safety without wasting human resources. This study, therefore, analyses the subjective alertness self-assessment, attentional performance (SA and SUA), sleep status, and in-flight rest of different numbers of pilot crews under the present conditions of the COVID-19 pandemic. It also explores the fatigue of crews under the new exempted organization model to provide theoretical support for pilot allocation, duty time, rest time, and alertness prediction in long-haul missions, which provide a scientific basis for flight safety and the effective use of human resources.

In summary, this study aims to examine whether there are differences in the fatigue levels and sleep quality of crews with different numbers of pilots by combining subjective and objective measurement methods. Furthermore, we investigate pilots’ sleepiness, fatigue, and alertness levels during different flight phases and discuss the possible influencing factors.

## 2. Materials and Methods

### 2.1. Participants

Among fifteen pilots who performed 2–4 intercontinental round-trip flights (Table 1), a total of 42 times were recorded. They each completed missions with different crew sizes: at least two 4-person (China–Canada non-exempt flights), 6-person (China–Australia exempt flights), and 8-person (China–USA exempt flights) crews, respectively.

The impact of departing time was minimized to the greatest extent possible, but it was impacted by the efficient use of employees and unexpected occurrences, such as airline delays, cancellations, and meltdowns. Four non-exempt pilots took off at around 14:00 in the afternoon with an 11-h outbound and 13-h inbound flight time, while six and eight exempt pilots took off after 23:00—a six-pilot flight with a 9-h round-trip and an eight-pilot flight with a 12-h outgoing and 15-h inbound flight time, respectively.

### 2.2. Procedures

**Figure 1:**
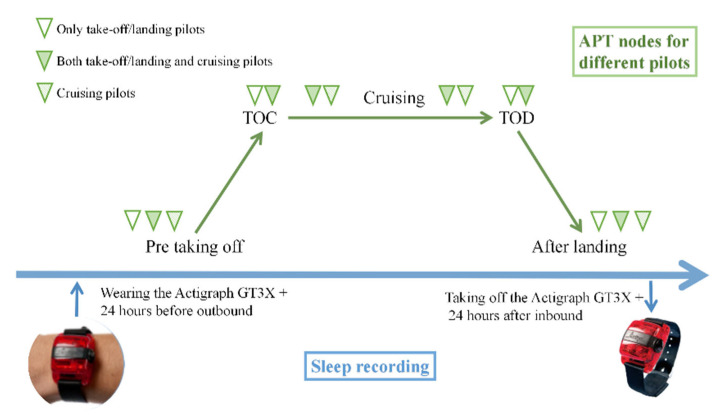
Data collection processes. TOC is the point when the aircraft reaches its initial cruising altitude, and TOD is the point at which the aircraft transitions from the cruise to landing phase of flight.

The pilot starts wearing the sleep detection bracelet 24 h before outbound takeoff and can take it off until 24 h after the end of the inbound flight. During the flight, pilots are asked to perform subjective self-assessment and attentional tasks before takeoff, before working, after working, and after landing. There are three possibilities for pilots’ schedules, as shown in Figure 1.

### 2.3. Measurements

#### 2.3.1. APT

**Figure 2:**
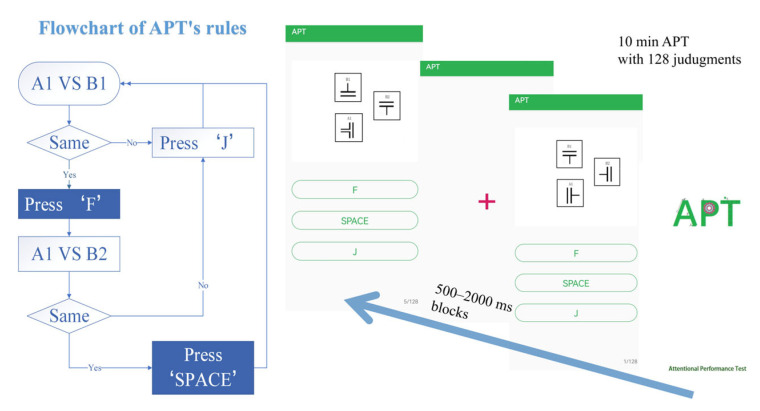
APT rules diagram.

A common experimental paradigm is the continuous performance test (CPT), in which subjects are asked to monitor the visual or auditory presentation of a single letter or number and respond when a target stimulus is presented [19]. The attentional performance test (APT) used in this study evolved from the CPT. As stimuli of APT, the symbols {╤╟ ╤ ╢╩╠ ╦ ╣} without semantic connotations could limit the practice effect, which enables more reliable sensing of the participant’s alertness and attention levels [20]. The APT uses HONOR ViewPad 6 (HONOR, Shenzhen, China, resolution: 2000 × 1200 pixels, screen size: 8800×5000 mm) to present simulations and record participants’ reaction times and answers (Figure 2). First, participants compare the symbols A1 and B1; they choose “F” if the symbols are the same and “J” if they are not. Subsequently, if the previous answer is the same (“F”), they are asked to compare symbols A1 and B2; they press “SPACE” when the symbols are the same and “J” if they are different. If the answer is “J,” they go back to the first step. Overall, the APT contained 128 judgments, with random intervals of 500–2000 ms blocks between stimulations. The duration of this task was around 10 min.

#### 2.3.2. Self-Report

The Karolinska Sleepiness Scale (KSS) was selected to evaluate pilots’ sleepiness [21]. The scores range from “1 = extremely alert” to “9 = very sleepy”. It is a widely used tool for quantifying alertness in alertness and sleep investigations. The KSS is a sensitive and reliable measure of sleepiness and has been validated against many physiological measures [22].

The Samn–Perelli Crew Status Check (SP) was selected to evaluate pilots’ fatigue [23]. The scores range from “1 = fully alert” to “7 = completely exhausted (unable to function effectively)”. The SP is widely used in crew fatigue studies and has high reliability and validity [24].

#### 2.3.3. Actigraph GT3X +

**Figure 3:**
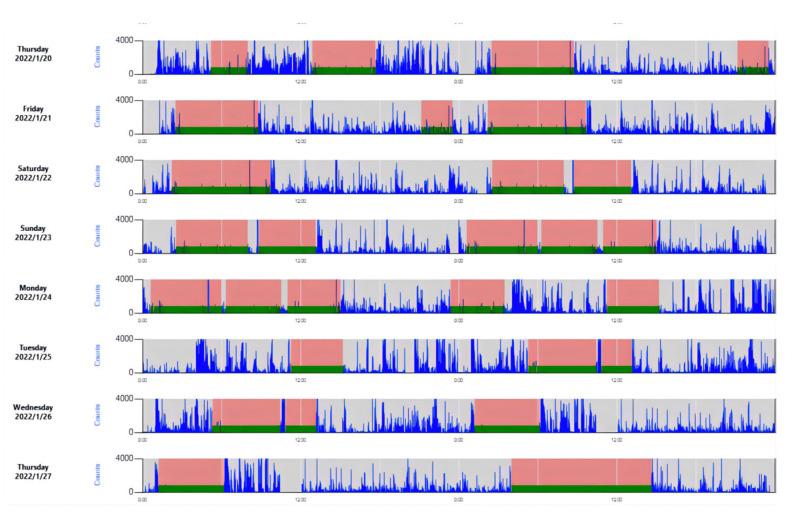
Sleep analysis example using ActiLife 6.13.4. The translucent red denotes sleep period, the green denotes sleep score indicator, and blue denotes activity counts.

Actigraph GT3X + recorders (Manufacturing Technology Inc. MTI, Pensacola, FL, USA) were used to record the somatic motion of pilots. It is a small (4.6 cm × 3.3 cm × 1.5 cm) and lightweight (19 g) triaxial acceleration monitor widely used in physical activity and sleep behavior studies [25], which is highly consistent with polysomnography to identify and monitor individual sleep–wake states [26]. The frequency is 30 Hz with a high-pass filter setting of 0.25 Hz and a low-pass filter setting of 2.5 Hz. They can be used continuously for 25 days on a full charge and can store 180 days of recorded data (4G). Information, including pilot number, bodily position, and start time of recording, was collected using ActiLife 6.13.4 software (Figure 3) and the sleep periods were auto-analyzed using the Cole–Kripke algorithm [27]. The following indices were used to evaluate sleep quality: sleep efficiency (SE, %), total sleep time (TST, min), wakefulness after sleep onset (WASO, min), number of awakenings (NOA), and average awakening length (AAL, min).

### 2.4. Analysis

The study contained two independent variables, more specifically, the first one was crew composition and the second one was flight phase, and the dependent variable was pilot’s fatigue level. The goal was to determine the interaction of different crew compositions and flight phases, and the variables alone influenced pilot fatigue and alertness in a statistically significant way. We utilized SPSS 26.0 software (IBM Corp., Armonk, NY, USA) to statistics, and we employed the two-factor mixed-design ANOVA to compare the attention efficiency (the reaction time and correctness of SA and SUA) and self-reported fatigue among different crew compositions and flight phases, which is usually used to compare the differences between groups that have been split on two “factors”, where one factor is a “within-subjects” factor and the other factor is a “between-subjects” factor [28,29]. The violation of sphericity was corrected by Greenhouse–Geisser. The Bonferroni (LSD) test was conducted to perform pairwise comparisons after a statistically significant interaction, and the effect size of factors and interactions were quantified by partial *η^2^*. Meanwhile, Kruskal–Wallis test was performed to test whether the differences in pilot number have statistically significant influences on each sleep indicator, APT, and self-assessment during the same phase (e.g., outbound and inbound for sleep, and pre-takeoff, pre-cruise, and post-landing for APT and self-report) [30]. At last, Spearman’s correlation coefficients were used to express the interdependency between study variables. The average RTs, correct rates, self-reported fatigue, and sleep indexes were analyzed using Spearman’s correlation coefficient to reflect the relationship among sleep quality, task performance, and self-assessment. A significance level of *p* < 0.05 was applied to all analytical methods, where *p* < 0.05 represents statistically significant differences existing between study variables.

## 3. Results

### 3.1. Sleep

Table 2 summarizes the results of sleep quality recorded by Actigraph GT3X +. In-flight and layover sleep was shorter and less efficient than before and after the mission, and the larger the crew size the longer the total sleep time available on the aircraft. Meanwhile, on the outbound trip, the traditional four-person crew slept shorter and less efficiently than on the inbound trip, while the exempted six-person or eight-person crews slept worse on the inbound trip than the outbound.

The sleep duration before outbound flights and after inbounding were overall more than 400 min. Additionally, the TST was significantly lower during layover abroad (around 350 min) than before and after the flight (*p* = 0.019), while the SE was also slightly lower without statistical variation. Pilots of all crew sizes’ TST and SE after a mission were able to regain the level of not statistically different from the pre-flight sleep.

During the flight, the TST of the crew was concentrated between 100 and 300 min, and only the eight-person crew sleeping more than 200 min during outbound and inbound missions had desirable SE. Significant differences were found in sleep on outbound flights with various crew sizes. In particular, the SE and AAL of four-pilot flights were lower (*p* = 0.015) and longer (*p* = 0.019) than others. Meanwhile, the four-pilot TST was the shortest, showing a significant difference to the six- (*p* = 0.007) and eight-pilot (*p* = 0.000) flights. Additionally, regarding inbound sleep or naps, pilots’ SE on the six-person flights was slightly lower than on the eight-person flights (*p* = 0.046). Conversely, the four-person crew exhibited a prolonged TST (*p* = 0.023) for the inbound trip compared to the outbound flight, with no statistically significant difference in SE. As the flight duration varies on different routes, the average in-flight TST per pilot on a flight per hour of travel was calculated (mean TST on the flight/total flight time of the flight). The four-person crew exhibited 11.2 min/h sleep for the outbound journey and 12.6 min/h for the inbound; the six-person crew, 26.4 min/h for the outbound and 17.9 min/h for the inbound; and the eight-person crew, 23.5 min/h for the outbound and 16.3 min/h for the inbound.

Overall, the eight-person crew sleeps for approximately 4–5 h on a flight, with an optimal sleep efficiency of approximately 95%. The return journeys for a six-person crew and the outbound trips for a four-person crew have short (around 160 min) and inefficient (around 89%) sleeping hours, and the sleep on the outbound flight of the six-pilot flight was statistically different from the inbound flight. The outbound sleep showed longer sleep duration (*p* = 0.03) and higher sleep efficiency (*p* = 0.02) than the inbound one.

### 3.2. Self-Report

The results indicated a high level of crew alertness in-flight with all KSS scores lower than 5 and an SP of around 2, regardless of crew size and exemptions. SP (2.01 ± 0.825) and KSS (2.56 ± 1.29) scores showed statistically correlation (*r* = 0.782, *p* = 0.000) overall. According to the result of the two-way mixed-design ANOVA (Table 3), the interaction between flight phase and number of pilots also demonstrated a statistically significant influence on KSS (*F* = 3.514, *p* = 0.000, *η*^2^ = 0.695) and SP (*F* = 3.645, *p* = 0.000, *η*^2^ = 0.703). The flight phase has a statistically significant independent effect on KSS (*F* = 5.663, *p* = 0.000, *η*^2^ = 0.786) and SP (*F* = 7.418, *p* = 0.000, *η*^2^ = 0.828); the number of pilots also has a statistically significant independent effect on KSS (*F* = 3.544, *p* = 0.041, *η*^2^ = 0.181) and SP (*F* = 5.983, *p* = 0.006, *η*^2^ = 0.272), with the most notable difference in SP (*p* = 0.002) and KSS (*p* = 0.015) for four and six pilots according to LSD.

Crew fatigue and sleepiness perceptions were markedly higher than before by the end of the mission. Additionally, pilots on takeoff and cruising duties showed a rapid upward trend in both KSS and SP, especially for inbound flights. Simple effect analysis indicated that four-pilot showed significantly different effects of KSS on flight phases (*F* = 4.424, *p* =0.002), according to the results of post hoc comparisons: outbound and inbound after landing > pre taking-off, pre-cruise; inbound TOC, and after taking-off and cruising > pre taking-off. Six-pilot (*F* = 5.147, *p* =0.001) also showed differences, according to the results of post hoc comparisons: outbound pre-cruise > inbound pre-cruise; outbound after cruising > after taking-off and cruising, TOD, and inbound pre-cruise; and inbound after taking-off and cruising, and TOD > pre-cruise. There were no statistically significant differences in eight-pilot (*p* = 0.051). For SP, four-pilot (*F* = 4.426, *p* =0.002) showed significantly different: inbound TOC, and after taking-off and cruising > pre taking-off; inbound TOC > pre-cruise. Six-pilot (*F* = 9.331, *p* = 0.000) showed differences: outbound TOC, pre-cruise, after cruising and TOD > inbound pre-cruising. The eight-pilot (*F* = 4.478, *p* = 0.011) also saw differences: outbound after taking-off and cruising, and inbound TOD > outbound TOD and pre taking-off. These results demonstrated that overall self-report fatigue of different flight phases is different among crew composition.

**Figure 4:**
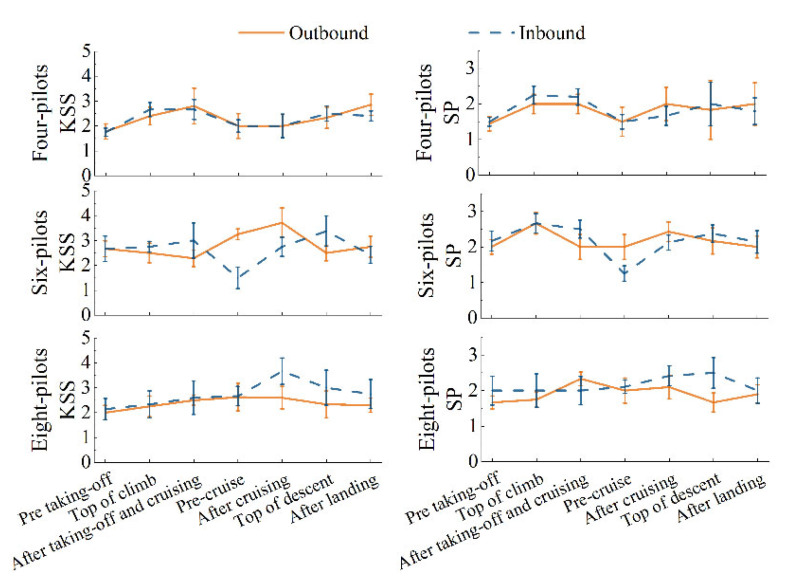
Subjective self-assessment of all patterns.

As shown in Figure 4, the subjective self-assessed fatigue of the four-person crew had the same trend for both the outbound and inbound missions without a statistical difference. The first peak occurred after taking off and cruising and a second peak after landing. Additionally, there was an increase in fatigue after cruising compared to pre-cruise, with a more pronounced SP scale. For the six-person crew, who were most obviously affected by the flight phase, pilots reported the highest KSS (3.71) after cruising during the outbound and SP (2.67) at the top of the climb. The KSS showed a slightly declining trend during the outbound takeoff period but increased during the inbound period. The SP values increased on takeoff and cruise but showed a slight decrease on landing. The self-reported fatigue of the eight-pilot crew was the lowest, especially in the outbound trip with KSS and SP at around 2. KSS rose slowly in the inbound journey, reached a maximum after cruising (3.66), and then decreased. The SP peaked after takeoff and cruising on the outward leg (2.33) and at the top of descent on the inward leg (2.5). Notably, the SP before takeoff was noticeably higher on the return trip than on the going trip in the eight-crew flight.

### 3.3. APT

Two-way mixed-design ANOVA showed that the interaction between flight phases and number of pilots shows a notable difference on SUA’s RT (*F* = 2.047, *p* = 0.007, *η*^2^ = 0.372), while they did not display a statistically significant influence on other indicators of attentional performance. The flight phase has statistically significant independent influences on SA’s RT (*F* = 5.532, *p* = 0.000, *η*^2^ = 0.615) and CR (*F* = 1.793, *p* = 0.049, *η*^2^ = 0.030); the number of pilots has a statistically significant influences on SUA’s CR (*F* = 5.682, *p* = 0.006, *η*^2^ = 0.166), as shown in Table 4.

There were fluctuations in attentional performance across flight phases and different pilot number. Overall, the pre-cruising RTs of six- and eight-pilot flights were the highest with a more stable trend on the inbound leg, and the four-pilot flight had a higher mean correct rate than the six- and eight-pilot crews. The interaction test showed significant results for both flight phase and crew composition of SUA’s RT, so the simple effect was analyzed. The four-pilot showed significantly different effects on flight phases (*F* = 5.788, *p* =0.000), according to the results of post hoc comparisons: pre taking-off > outbound pre-cruise and inbound after cruising. The six-pilot (*F* = 2.627, *p* = 0.008) showed significantly different: outbound pre-cruise > other outbound phases and inbound pre taking-off and pre-cruise. The eight-pilot (*F* = 3.022, *p* =0.003) also saw a significantly different: outbound pre-cruise > outbound TOD and after landing and inbound TOC. The interaction tests for flight phase and number of pilots did not yield significant results in CR and RT of SA, and CR of SUA, so the main effect was analyzed. The influence of SA’s RT (*F* = 3.212, *p* = 0.001) varies significantly among flight phases, while the CR of SUA showed a statistical difference between pilot number (*F* = 5.682, *p* = 0.006). According to the multiple comparison, the RT before outbound (M = 1665.131 ms) and pre-cruising (M = 1723.317 ms) were significantly longer than that after cruising (M = 1470.301 ms) and top of descent (M = 1301.763 ms). Meanwhile, the SUA of four-pilot (M = 0.974) showed the highest mean CR, followed by six-pilot (M = 0.975) and eight-pilot (M = 0.960).

**Figure 5:**
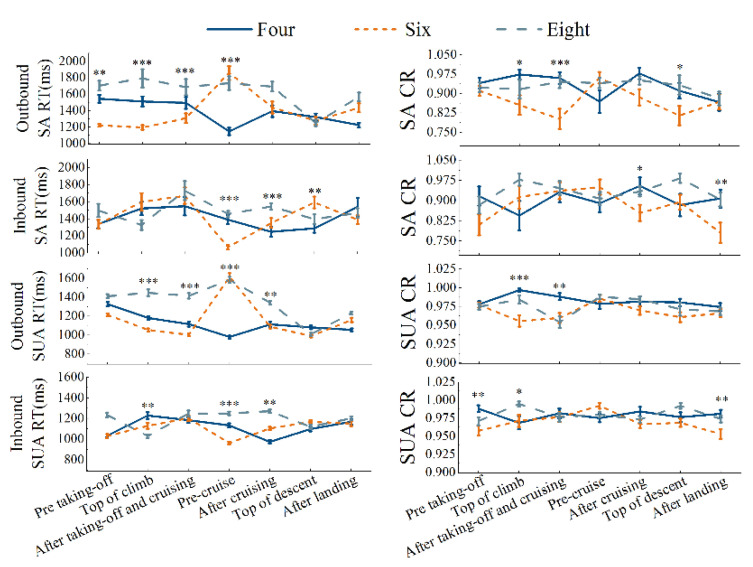
RT and CR of SA and SUA. * *p* < 0.05; ** *p* < 0.01; *** *p* < 0.001. SA denotes selective attention; SUA denotes sustained attention. RT denotes reaction time; CR denotes the correct rate.

In addition, the RT of the four-person crew SUA differed from the flight variation trend of the six- and eight-pilot crews. On the outbound journey, the longest RT was found for the eight-pilot crew (SA: 1532 ± 62 ms; SUA: 1348 ± 23 ms), followed by the six-pilot crew (SA: 1390 ± 51 ms; SUA: 1160 ± 20 ms), and the shortest RT was found for the four-pilot flights (SA: 1376 ± 53 ms; SUA: 1122 ± 21 ms), while no clear difference in the mean RT was shown on inbound flights among crew sizes (SA around 1400 ms; SUA around 1100 ms). The CR of SUA (around 0.975) was more stable and higher than SA (around 0.9), especially on the inbound mission, while the RT of SUA fluctuated at 1200 ms shorter than SA at approximately 1400 ms (Figure 5).

Overall, there was a marked extension of RT after the cruise, TOD, and landing on the outbound flight, and the attentional performance was somewhat reduced from TOC on the inbound. During the outbound flight phase, RTs of SA after cruising (*p* = 0.49) and TOD (*p* = 0.013) were different from those pre-takeoff, and SUA’s RT showed a significant difference from pre-takeoff after cruising (*p* = 0.16), TOD (*p* = 0.002) and after landing (*p* = 0.33), respectively. On inbound flights, the RT of SA increased after cruise compared (*p* = 0.46) to pre-cruise, and CR after landing (*p* = 0.01) was statistically different from pre-mission. Additionally, the SUA’s CR was stable for each flight phase during the inbound journey, while the RT of TOC (*p* = 0.017) and after cruising (*p* = 0.08) was different from pre-mission.

For SA, the RT of the four-person crew was more stable during the outbound mission, and the CR reached its lowest value (0.87) during pre-cruise. Compared to pre-cruise, both RT and CR showed an increasing trend after cruise, and RT was longer after takeoff and landing in the inbound leg than before. The six-person crew showed a significant decreasing trend in CR during takeoff, while it exhibited an extended RT during landing. On the inbound flight, RT increased during the takeoff, cruising, and landing phases, while CR decreased after cruise and landing. Finally, the eight-passenger crew had a more stable SA during the outbound and inbound phases, with a shorter RT and a lower CR during the departing landing phase.

For the SUA, the RT during the outbound mission of the four-person crew was constantly shorter, and the CR was at a maximum TOC (0.99). Both RT and CR increased after cruising compared to pre-cruise. On the inbound leg, RT became longer after takeoff and landing than before, while RT was shorter after cruising than before. The six-person crew showed a slight decrease in RT and CR during takeoff, while RT and CR increased during landing. On the inbound flight, RT rose during the takeoff and landing phases. Post-cruise SUA was significantly worse, showing a decrease in RT and a rise in CR. The pre-cruising and landing CRs were lower than before at 0.96 and 0.95, respectively. Finally, the eight-person crew had a trough in the CR during the outbound after takeoff and cruising at 0.95. The RT fluctuated at around 1400 during the takeoff and cruising phases, and around 1200 ms during landing. On the return trip, CR showed slightly higher peaks at TOC and TOD, 0.994 and 0.992, respectively.

### 3.4. Subjective and Objective Correlation

To investigate whether there was certain relationship among sleep, subjective perceptions, and attentional performance, they were tested separately to determine the correlation. Subjective self-assessment results do not notably correlate with performance in the SA and SUA. The results showed that KSS and SP values were negatively correlated with the number of awakenings and wakefulness after sleep onset in sleep. Simultaneously, the correlation between subjective fatigue and sleep quality was more significant in the six-pilot crew than in the four- and eight-pilot groups.

Overall, the KSS and SP values were positively correlated with RT and negatively correlated with CR. The RT of SA was relatively more clearly correlated with KSS and SP (*r* = 0.116, *p* = 0.000; *r* = 0.119, *p* = 0.000) than SUA (*r* = 0.065, *p* = 0.000; *r* = 0.065, *p* = 0.000). Meanwhile, KSS and SP scores were more highly negatively correlated with SA correctness (*r* = −0.057, *p* = 0.00; *r* = −0.071, *p* = 0.000) than SUA (*r* = −0.029, *p* = 0.00; *r* = −0.039, *p* = 0.000).

Table 5 demonstrates the correlation between the self-assessment results and sleep indices for different crews. SP was negatively correlated with TST, NOA, and WASO in all size groups, with higher SP values for shorter TST, which was most pronounced in the six-pilot crews. The results also indicated that SE correlated with SP in only six of the groups (*r* = 0.302). For KSS, it was the most dramatically correlated with NOA, with a higher KSS correlated with less NOA, which was still most noticeable for six-person crews. Additionally, the six-person group showed a significant negative correlation between KSS and TST (*r* = −0.624). However, AAL was not statistically correlated with subjective drowsiness nor with fatigue.

## 4. Discussion

### 4.1. Sleep

The pilots were well rested before and after flights with around 7 h sleep and an SE greater than 90% without a significant difference in sleep after missions between crew sizes. The TST was reduced by around 100 min at abroad layover than before mission, which supports H2. In the flight, the larger the crew size, the longer TST and the more desirable the SE, most notably in the outbound tasks. The four-person crew had the shortest TST available on the outbound of approximately 2 h, with the lowest SE. The six- and eight-pilot groups had a higher SE at approximately 95% with TST of around 4–5 h, which agrees with H1 to a certain extent.

However, the flight duration and takeoff time also have an impact on the quality of sleep [3]. A study found that for each additional hour of flight time, pilots slept an average of 12.3 min more [31]. To avoid this effect, we calculated the TST available during the same working time per person. Specifically, the average worked hour per person was first calculated by dividing the number of flight hours by the number of pilots on the flight. Then, the average TST of pilots was divided by the number of the worked hour per person. The results showed that TSTs of 188.2, 158.8, and 44.95 min were obtained for the eight-, six-, and four-man crews, respectively, during the outbound trip, while 130.6, 107.8, and 50.43 min during the inbound trip. In conclusion, the larger the crew size, the longer the available rest time. Eight-pilot crews slept on average around 50 min more than six-person crews, and the outbound trip was 144 min longer than for four-person crews.

We also noticed that the outbound TST was longer for the six- and eight-person groups than the inbound journey, whereas the four-person group experienced the opposite. This might due to a combination of the pilots’ biorhythms and the takeoff time [12]. On the outbound flights, both the six- and eight-person crews left at night, and the time when they were allowed to rest aligned with the biological clock’s nocturnal sleep cycle. As a result, some pilots could obtain a good night’s sleep on the evening mission with a longer TST. It may also be the case that the traditional non-exempt four-pilot crew had a full night’s rest at the end of the outbound mission, which helped them recover from fatigue, and a less psychologically stressful inbound flight, resulting in a better-quality sleep than on the outbound flight.

In conclusion, when adding pilots to the exempt operation, the sleep on inbound flights was slightly worse than on outbound flights, so airlines should take measures, such as improving the resting environment on board, optimizing the passing procedures, and encouraging takeoff and landing crews to take naps during short overlays, to keep pilots of exempt flights at a better level of alertness on the return trip. For traditional non-exempt flights, airlines should create a schedule to ensure that pilots have been adequately rested after their last duty; crews should take a nap before takeoff and pay attention to their fatigue condition, introducing caffeine or melatonin intake if necessary.

### 4.2. Self-Reported Fatigue

The crew maintained positive alertness during the flight, according to subjective self-assessment. Prolonged duty periods lead to an increased workload, which in turn causes greater feelings of fatigue [26]. A layover break could recover and alleviate the pilots’ fatigue before the inbound flight, as evidenced by the lack of statistical difference between the four-person crew on the pre taking-off of outbound and inbound trips. There was the same trend in fatigue and sleepiness perception between the four-person crew on the outbound and inbound trips. However, the fatigue levels of the exempted six- and eight-person crews before the inbound flight were elevated compared to the outbound flight.

Overall, the level of alertness after takeoff, cruise, and landing decreased in comparison to the pre-flights; the smaller the crew size, the more obvious this was, and H3 was verified. According to existing studies, there are significant differences between flight phases [32]. On multiple takeoff and landing days, crews feel greater fatigue than on single-segment duty days [33]. We also found that the four-person crew’s drowsiness grew notably after the takeoff and landing phases, but the six-person crews’ drowsiness peaked after the cruise, while the level of sleepiness of the eight-person crew did not differ statistically across flight phases. This might be due to differences in the number of crews, leading to variations in their duties; for example, the four-pilot crews on non-exempt flights that are responsible for takeoff and landing also need to complete part of the cruise duties, with a longer working duration, which leads to a significant increase in fatigue and sleepiness as they perform takeoffs and landings.

The SP and KSS were substantially connected to the pilots’ fatigue and sleepiness levels in this study, and a shorter TST was associated with greater sleepiness and fatigue. Another study found that subjective sleep quality was related to the level of exhaustion [34]. However, the association between subjective self-assessment and attentional performance contradicts previous research findings [35]. We also discovered that sleep status had the highest connection with subjective reports in six-person crews. The four-pilot crew had a short rest in-flight, while the eight-person crew had a long duty time, so they may overestimate their own fatigue level due to mental stress. There are two plausible explanations for this result: the pilots did not respond accurately, and there was a difference between subjective perception and objective attentional performance. First, creating a safe cultural environment by emphasizing the hazards it can cause in fatigue education can promote fatigue reporting and improve its accuracy [36]. Second, frequent shift work may reduce their subjective sleepiness sensitivity [37]; therefore, focusing on this group can reduce the risk of unsafe events due to excessive or insufficient self-rated fatigue.

In conclusion, managing crew fatigue through limited self-reported results was inaccurate, and airlines should establish and continuously improve fatigue risk management systems to better mitigate human-induced risks. Eight-person crews experienced less fluctuation in fatigue and sleepiness during different phases of the flight than the four- and six-person crews, which was caused by the increased number of rotations. This provides a new approach to pilot shifts on traditional flights, whether it is longer continuous work or shorter multiple work sessions, to better help alleviate fatigue, and the scientific shift of pilots in different phases during flights warrants further study.

### 4.3. Attentional Performance

Crew attention performance varied among flights with different numbers of pilots and different fling phases, more specifically, RT fluctuated significantly across flight phases, while CR varied across flights with different numbers of pilots. Simultaneously, RTs of four-pilot crews are more susceptible to flight phases than those of six and eight. Overall, SA has more pronounced fluctuations, longer RTs, and lower CRs compared to SUA, which requires further action to improve its stability. SA was also more notably correlated with the self-assessment of fatigue and more sensitive to the quality of sleep than SUA.

The layover sleep could slightly recover attention performance, and exempted flights had slightly longer RTs and lower CRs than the traditional pilot before inbound departure. After the inbound landing, the RT of all crews were longer than that of the outbound landing, with lower CR supporting H3; in particular, the four-person crew had the longest RT for SA, and the eight-person crew had the lowest CR. There was a significant drop in pilot alertness levels after long working hours, even when they had the opportunity to take an onboard or layover break.

The six- and eight-person crews had longer RTs before the outbound cruising, probably due to being at the window of circadian low (3:00–6:00) and having a poor quality of rest during takeoff, which agrees with H4 [1]. Meanwhile, the level of alertness of a traditional four-person crew’s cruising pilots was reduced during long working hours. The SA and SUA performance of the four-person crew slightly deteriorated after the outbound cruise compared to pre-cruise. For augmented crews, the pilots responsible for takeoff and landing could enjoy a full and continuous rest, while the broken sleep of the cruising crew may lead to poor alertness during cruising. Therefore, the drowsiness and alertness of pilots who are responsible for long and continuous cruise operations in the future warrant further attention and could be controlled in advance using the fatigue risk management system [38] or a biomathematical model [39].

In conclusion, the effect of different crew sizes on takeoff and landing pilots’ SA and SUA performance was not significantly different, while pilots responsible for cruise missions may have performance fluctuations due to the quality and length of their in-flight sleep and time of day [40]. Meanwhile, the sleep and alertness of the crew responsible for different phases of flight and different in-flight shift schedules should be further investigated in future studies.

### 4.4. Limitations

This study has the following limitations: First, it did not compare the individual differences of pilots. Although existing studies indicate that gender, age, and position do not have a significant effect on sleep condition during flights [41], pilots’ social relationships and family members’ status may have impacted their emotions and motivation [20]. Second, due to the influence of the pilot isolation policy during the COVID-19 outbreak, sleep data were only collected from the crew the day before takeoff and the day after the flight, so there was no detailed analysis of the post-flight sleep recovery. Meanwhile, the flights collected in this study did not depart at the same time due to delays, which might influence the fatigue and alertness of the crew due to circadian rhythms. Finally, multi-modal fatigue measurement methods were not used due to the limited space of the cockpit, making it difficult to identify short naps. Future studies could combine pilots’ inherent characteristics, such as personality and sleep type, with their sleep and circadian rhythm during exempt flights to improve the analysis of each influencing factor and provide more accurate data to improve the exemption policy.

## 5. Conclusions

This study showed that expanding crew sizes to complete direct intercontinental round-trip flights would not negatively impact pilot alertness levels and would be desirable in COVID-19 standing management. First, larger crew sizes provide pilots with more in-flight relaxation or sleep time. Particularly, pilots in crews of six and eight were able to receive long periods of continuous sleep, whilst crews in charge of cruising should be encouraged to sleep before takeoff to improve their alertness. Second, when the pilots slept on layovers abroad, with considerable time zone variations, their sleep duration was significantly reduced due to jet lag. However, personnel on exempted flights showed decreased subjective and objective alertness before inbound flights compared to regular international flights, with no significant difference at the end of the round trip. Providing a better in-flight resting environment and further optimizing overseas layover procedures will contribute to improved fatigue relief and operation safety of the new long-haul flight model in the future. Additionally, the crew’s SA and SUA showed poor nighttime comfort, especially for pilots who just started working at WOCL. During different phases of flight, SA and SUA differed among pilots with varying crew sizes, and SA was more susceptible to fatigue. These findings indicated that the four-pilot crew’s in-flight sleep should be given more attention than others and that the exemption of in-flight shift schedules should be adjusted to ensure that cruising pilots have enough time to recuperate. Furthermore, pilots’ safety education and environment require improvement, particularly encouraging proactive tiredness reporting and self-health monitoring. Simultaneously, aircrew flying missions that violate their biological clocks and base circadian rhythms should be seriously considered.

## Figures and Tables

**Table 1:** Sample sizes.

Pilots Number	Outbound Takeoff Time	Inbound Takeoff Time	Total (Times)
Four-pilots non-exempt	13:00–15:00	14:00–16:00	8
Six-pilots Exempt	23:00–01:00	10:00–12:00	18
Eight-pilots Exempt	23:00–01:00	14:00–16:00	16
Total (times)			42

Times are in the base time zone.

**Table 2:** Sleep quality in different crew sizes.

Pilots Number	Pattern	TST (min)	SE (%)	WASO (min)	NOA (times)	AAL (min)
Four	Before outbound	400.5 ± 84.2	91.6 ± 3.0 **	40.7 ± 12.4 **	20.5 ± 6.7	2.31 ± 0.5 *
	Outbound	123.6 ± 22.3 ***	89.5 ± 6.7 *	14 ± 8.4	5.7 ± 2.1	2.5 ± 1.3 *
	Layover	327.3 ± 91.3 *	90.4 ± 5.1	36.4 ± 29.3	14.9 ± 6.8	2.3 ± 0.9
	Inbound	163.9 ± 51.7	92.0 ± 5.9 *	14.3 ± 11.8	6.0 ± 4.8	2.1 ± 1.4
	After inbound	454.5 ± 122.4	93.3 ± 3.7	32.1 ± 18.8	15.1 ± 9.7	1.9 ± 1.6
Six	Before outbound	419.1 ± 94.1	90.1 ± 3.6 **	47.4 ± 22.2 **	24.9 ± 12.9	2.4 ± 1.6 *
	Outbound	238.2 ± 59.2 ***	94.0 ± 5.2 *	13.6 ± 8.8	6.8 ± 3.9	1.9 ± 0.9 *
	Inbound	161.7 ± 49.2	89.9 ± 3.8 *	19.5 ± 12.0	7.1 ± 4.0	2.9 ± 1.9
	After inbound	419.6 ± 94.1	91.3 ± 4.1	39.5 ± 18.9	19.3 ± 9.4	2.2 ± 1.5
Eight	Before outbound	406.2 ± 112.2	93.9 ± 4.2 **	26.9 ± 19.8 **	15.5 ± 11.1	1.9 ± 0.9 *
	Outbound	282.3 ± 104.8 ***	95.9 ± 3.4 *	13.6 ± 12.6	8.4 ± 7.1	1.43 ± 0.83 *
	Inbound	245.0 ± 135.4	94.8 ± 4.9 *	11.7 ± 9.5	5.2 ± 3.4	1.9 ± 1.4
	After inbound	450.0 ± 103.4	93.6 ± 3.9	32.0 ± 23.0	16.0 ± 11.2	1.9 ± 0.9

* *p* < 0.05; ** *p* < 0.01; *** *p* < 0.001. All data are expressed as the mean ± SD. TST denotes total sleep time; SE denotes sleep efficiency; WASO denotes wakefulness after sleep onset; NOA denotes the number of awakenings; AAL denotes average awakening length.

**Table 3:** Analyze results of the subjective self-assessment.

	Indicators	*F*	*p*	*η* ^2^
A (Flight phase)	KSS	5.663	0.000	0.786
	SP	7.418	0.000	0.828
B (Number of pilots)	KSS	3.544	0.041	0.181
	SP	5.983	0.006	0.272
A × B (Interaction)	KSS	3.514	0.000	0.695
	SP	3.645	0.000	0.703

**Table 4:** Analyze results of the attentional performance.

	Indicators	*F*	*p*	*η* ^2^
A (Flight phase)	SA CR	1.793	0.049	0.030
	SA RT	5.532	0.000	0.615
	SUA CR	1.669	0.102	0.030
	SUA RT	6.901	0.000	0.666
B (Number of pilots)	SA CR	2.008	0.144	0.066
	SA RT	1.706	0.191	0.056
	SUA CR	5.682	0.006	0.166
	SUA RT	2.323	0.107	0.075
A × B (Interaction)	SA CR	0.991	0.488	0.219
	SA RT	1.380	0.153	0.285
	SUA CR	1.216	0.280	0.410
	SUA RT	2.047	0.007	0.372

SA denotes selective attention; SUA denotes sustained attention; RT denotes reaction time; CR denotes the correct rate.

**Table 5:** Correlation between self-assessment and sleep.

Pilots Number	Self-Report	TST	SE	WASO	NOA	AAL
Four	KSS	−0.182	0.085	−0.357 **	−0.381 **	0.016
	SP	−0.276 *	0.063	−0.451 **	−0.490 **	−0.04
Six	KSS	−0.624 **	0.176	−0.494 **	−0.519 **	−0.049
	SP	−0.641 **	0.302 **	−0.596 **	−0.644 **	−0.033
Eight	KSS	−0.197	0.031	−0.149	−0.233 *	0.104
	SP	−0.377 **	0.114	−0.283 *	−0.338 **	−0.012

* *p* < 0.05; ** *p* < 0.01. TST denotes total sleep time; SE denotes sleep efficiency; WASO denotes wakefulness after sleep onset; NOA denotes the number of awakenings; AAL denotes average awakening length. KSS denotes the Karolinska Sleepiness Scale; SP denotes the Samn–Perelli Fatigue Scale.

## Data Availability

The datasets generated and/or analyzed during the current study are not publicly available due to the confidentiality principle of private information of participants. However, they are available from the corresponding author on reasonable request.
